# Supplementary material for: DMRforPairs: identifying Differentially Methylated Regions between unique samples using array based methylation profiles
Source: BMC Bioinformatics. 2014 May 15;15:141. doi: 10.1186/1471-2105-15-141 (PMC4046028; doi:10.1186/1471-2105-15-141)
Supplement: Additional file 2 — DMRforPairs output for the comparison of A431-MCF7 and NA17018-NA17105. Please start from the HTML files in each folder. Available via the BMC Bioinformatics website. [file 1471-2105-15-141-S2.zip › 1394847754114233_MOESM2_ESM/A431_MCF7/figures/11789.pdf]

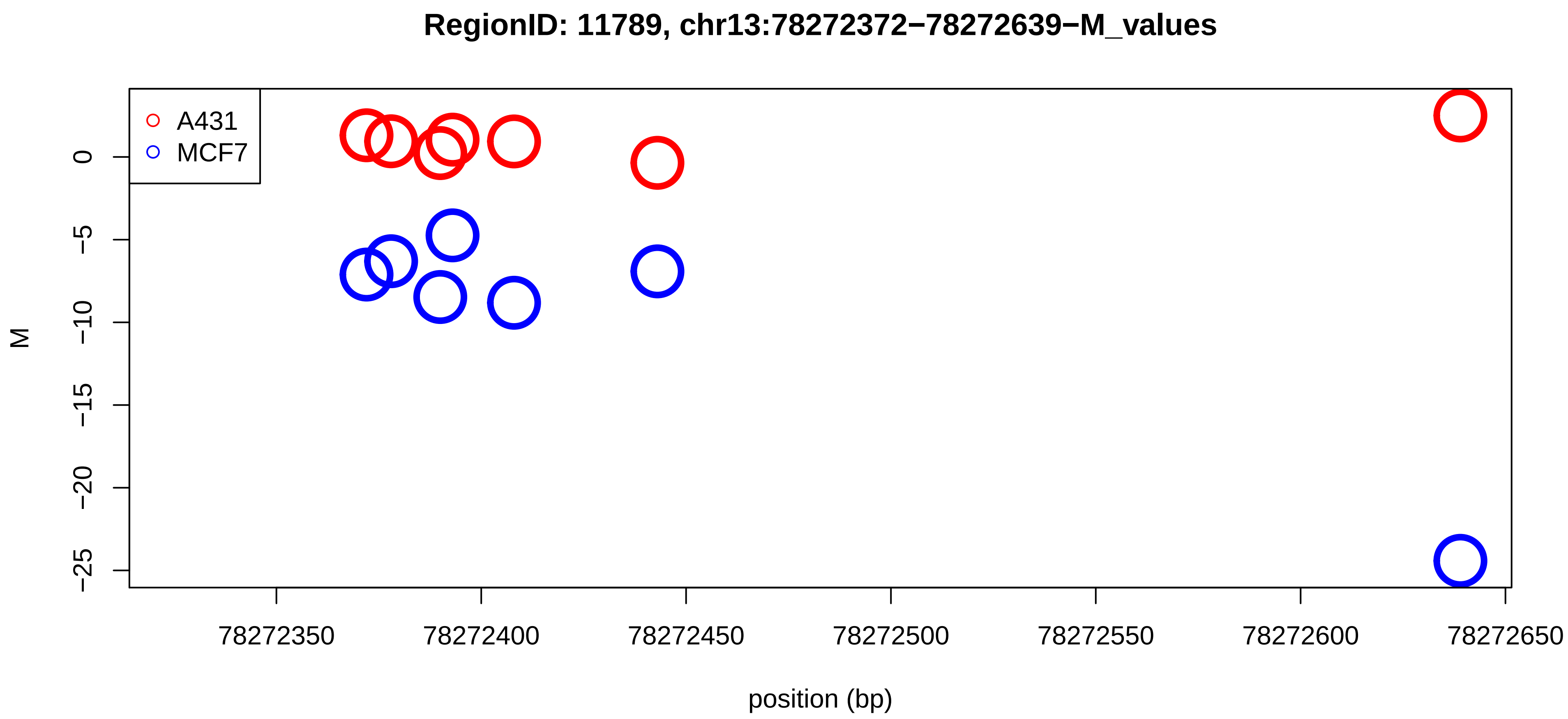

RegionID: 11789, chr13:78272372−78272639−M_values
A431
MCF7
M
0
−5
−10
−15
−20
−25
78272350
78272400
78272450
78272500
78272550
78272600
78272650
position (bp)

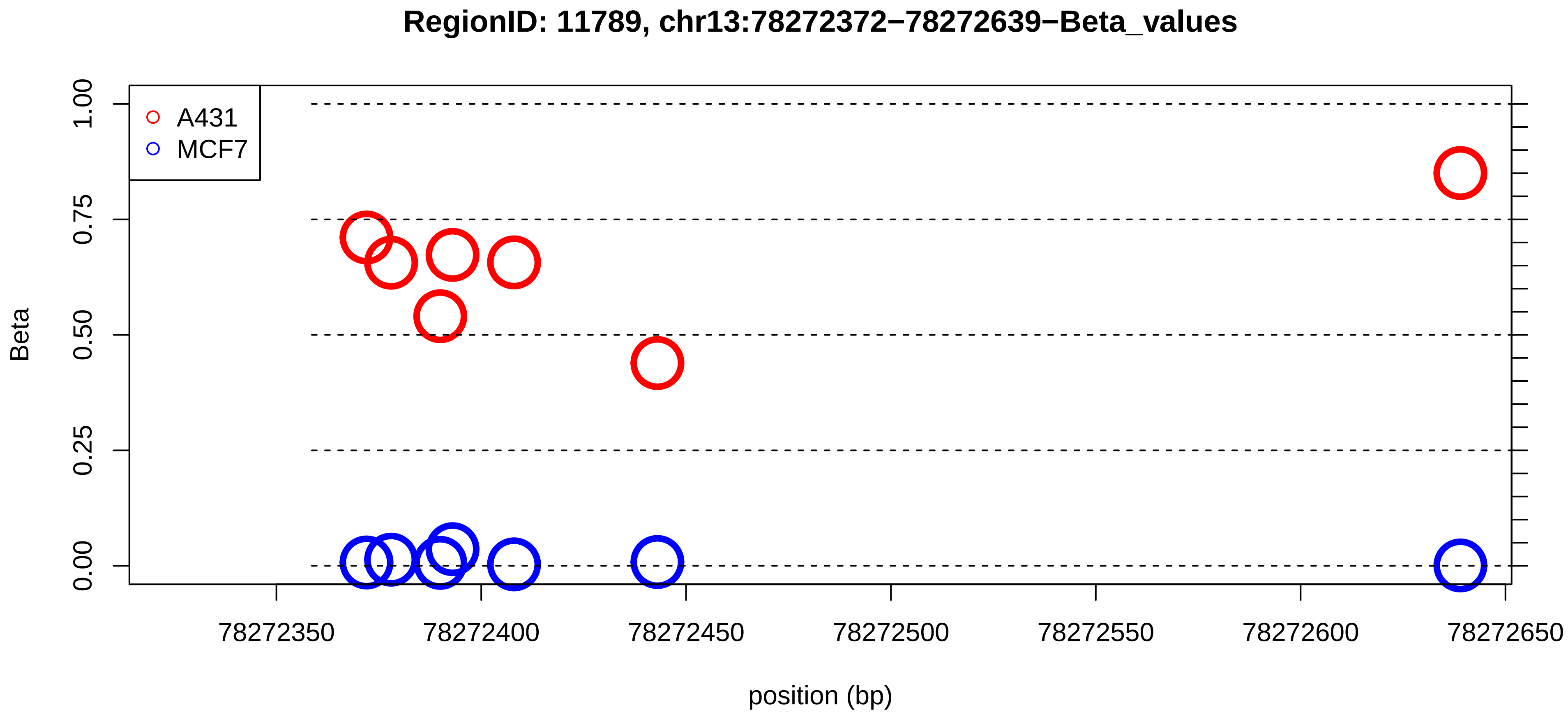

RegionID: 11789, chr13:78272372−78272639−Beta_values
A431
MCF7
Beta
0.00
0.25
0.50
0.75
1.00
78272350
78272400
78272450
78272500
78272550
78272600
78272650
position (bp)

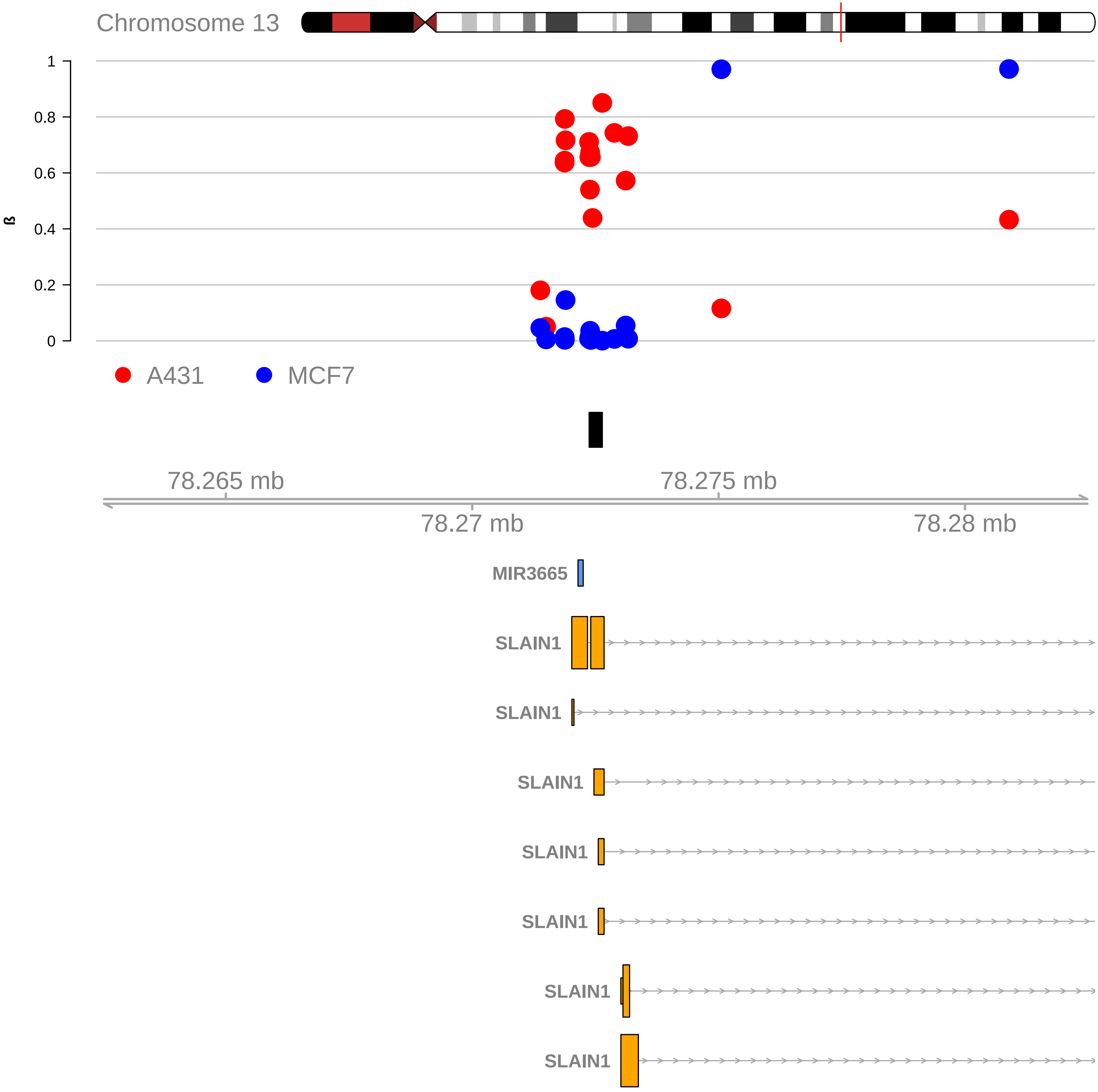

Chromosome 13
1
0.8
0.6
0.4
0.2
0
ß
A431
MCF7
78.265 mb
78.275 mb
78.27 mb
78.28 mb
MIR3665
SLAIN1
SLAIN1
SLAIN1
SLAIN1
SLAIN1
SLAIN1
SLAIN1
